# Surgical treatment of injection-induced rectus femoris muscle contracture in a child: A case report

**DOI:** 10.1016/j.ijscr.2024.109835

**Published:** 2024-05-31

**Authors:** Qusai Razzouk, Mohammed Al-mahdi Al-kurdi, Mohammed Diab, Mohamed Khair Kajjan, Hani Alloush

**Affiliations:** a Faculty of Medicine, University of Aleppo, Aleppo, Syrian Arab Republic; b Department of Orthopedic Surgery, Aleppo University Hospital, Aleppo, Syrian Arab Republic

**Keywords:** Quadriceps contracture, Injection-induced, Rectus femoris, Quadriceps Plasty, Orthopedic surgery, Case report

## Abstract

**Introduction and importance:**

Quadriceps contracture, characterized by the shortening of the quadriceps muscle and reduced knee flexion, poses challenges in daily activities. The etiology of this condition includes congenital, traumatic, infective, or iatrogenic factors.Treatment typically involves surgical intervention, with various techniques described in the literature. Differentiating between isolated rectus femoris contracture and combined rectus and quadriceps contractures is crucial for appropriate management.

**Case presentation:**

A 14-year-old female presented with gait disturbance and limitations in sitting and squatting due to rectus femoris contracture secondary to repetitive intramuscular injections. Physical examination revealed restricted knee flexion and positive Ely's test. The patient underwent rectus femoris lengthening (RFL) surgery, resulting in improved knee flexion and hip extension. Postoperatively, early mobilization and physiotherapy were initiated, leading to complete recovery with no complications during a three-year follow-up.

**Clinical discussion:**

Quadriceps femoris muscle contracture in childhood can result from congenital factors or acquired causes such as injections, trauma, infections, or ischemia. In Syria, injection-induced contractures are prevalent due to widespread intramuscular drug administration. Differentiating between isolated rectus femoris contracture and combined quadriceps contracture is crucial for treatment selection. Surgical intervention, such as rectus femoris lengthening using the Z-plasty procedure, yields favorable outcomes. Postoperative physiotherapy is essential. Incision necrosis is a common complication, mitigated by careful incision placement.

**Conclusion:**

Injection-induced rectus femoris contracture is common in children due to repeated thigh injections. Healthcare providers should consider alternative administration sites and routes to prevent contractures.

## Highlights

• Quadriceps Contracture muscle can be congenital or acquired.
• Injection-induced rectus femoris contracture muscle is common in children due to thigh injections.
• Quadriceps Contracture muscle Presents as knee stiffness with limited flexion, diagnosed via physical exams like Ely's test.
• Z-plasty lengthening of rectus femoris muscle effectively increases muscle length, facilitating early mobility and reducing recurrence risk.
• Healthcare providers should use methods other than intramuscular injection to administer medications.

## 1 Introduction

Various causes can lead to quadriceps contracture, a condition in which the quadriceps muscle is shortened [1]. The main symptom is reduced knee flexion, which interferes with sitting, squatting, or running. Lloyd Roberts, et al. first documented this condition in the literature, reporting six cases that underwent distal quadricepsplasty, but only three of them had satisfactory results at the final follow-up [2]. The literature has been inconsistent about quadriceps contracture, which can be congenital, idiopathic, or associated with arthrogryposis, or it can result from trauma, infection, or muscular injections [2,3]. The exact cause of the condition is still unknown, but it is clearly related to multiple muscular injections [2,4]. The contracture can affect one or more parts of the muscle [1]. It can appear as isolated rectus femoris contracture or combined rectus and quadriceps contractures, and it is important to differentiate between these two forms as they need different treatments. The isolated rectus contracture is best treated proximally, while the combined quadriceps form is more effectively released distally [2,5]. The quadriceps femoris is the main active knee extensor, so its contracture leads to extension contracture of the knee, resulting in a stiff extended knee that cannot flex enough. Other factors that can cause stiff extended knees are extra-articular conditions and adhesions of the capsule and synovial membrane [1]. The main treatment for this condition is surgery, except for mild cases where the knee flexion is more than 90^0^. The surgical technique that releases the adhesions and contractures in the quadriceps muscle is called “QUADRICEPS PLASTY”. There are various methods of quadricepsplasty described by different authors [1]. Early-stage proximal release and late-stage distal release are the suggested treatments [4]. We present a case of a female patient with rectus femoris contracture secondary to recurrent intramuscular injections in the right thigh. She underwent surgical rectus femoris lengthening (RFL) followed by postoperative physiotherapy. This manuscript was prepared by the SCARE 2023 guidelines [6].

## 2 Case presentation

A 14-year-old girl attended the ortho-pediatric clinic with complaints of gait disturbance and difficulty in sitting and squatting. No congenital anomalies were detected in the musculoskeletal system or other systems at birth. The clinical history revealed that she had received multiple intramuscular injections in the anterior region of the right thigh.

**Fig. 1:**
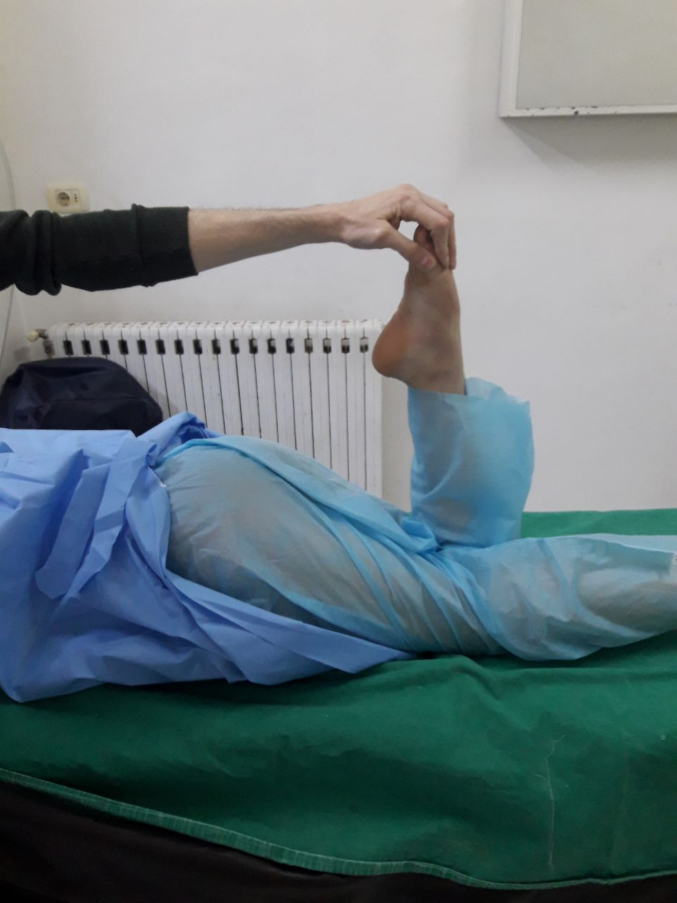
Ely's test.

**Fig. 2:**
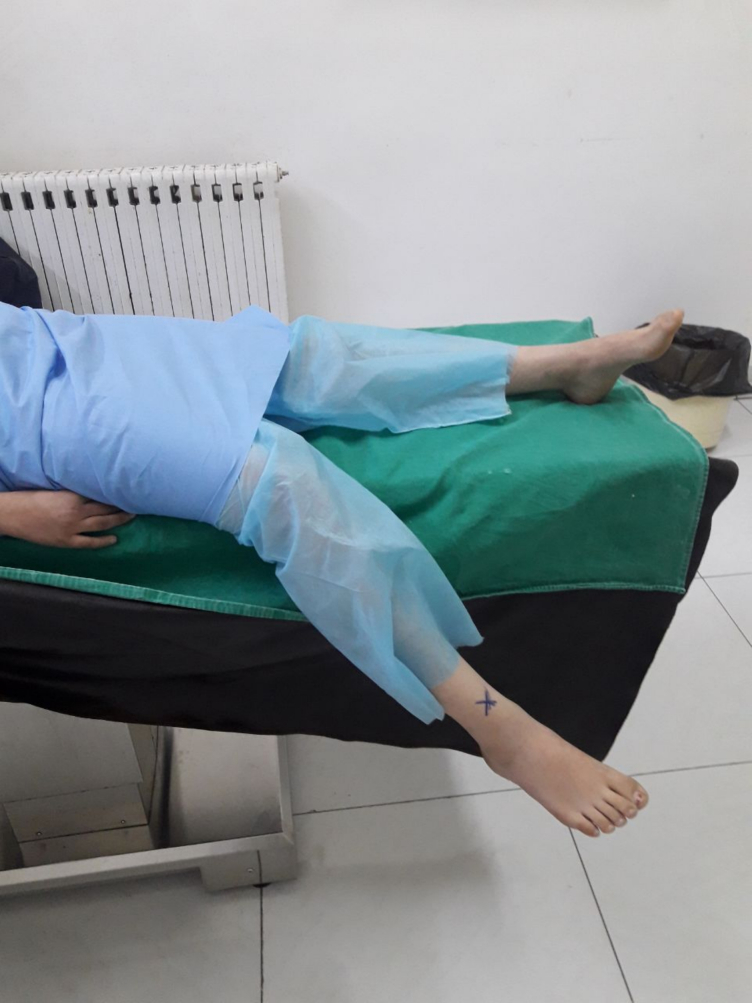
Test which the Patient seated, partially lowered affected limb.

On physical examination, she had restricted knee flexion at 45° with a hip extension due to contracture of the rectus femoris muscle. The Ely's test was positive Fig. 1. Furthermore, another test was performed in which the patient was instructed to sit on the edge of the examination table and lower her affected extremity off the table edge, which she could only partially accomplish Fig. 2. Other tests were normal and no other disorders were observed.

**Fig. 3:**
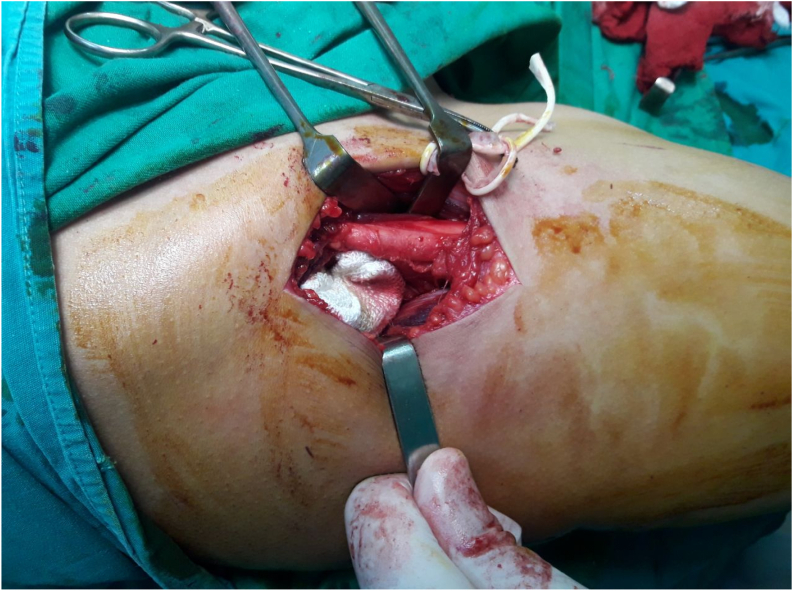
The rectus femoris muscle of the quadriceps femoris muscle before lengthening.

**Fig. 4:**
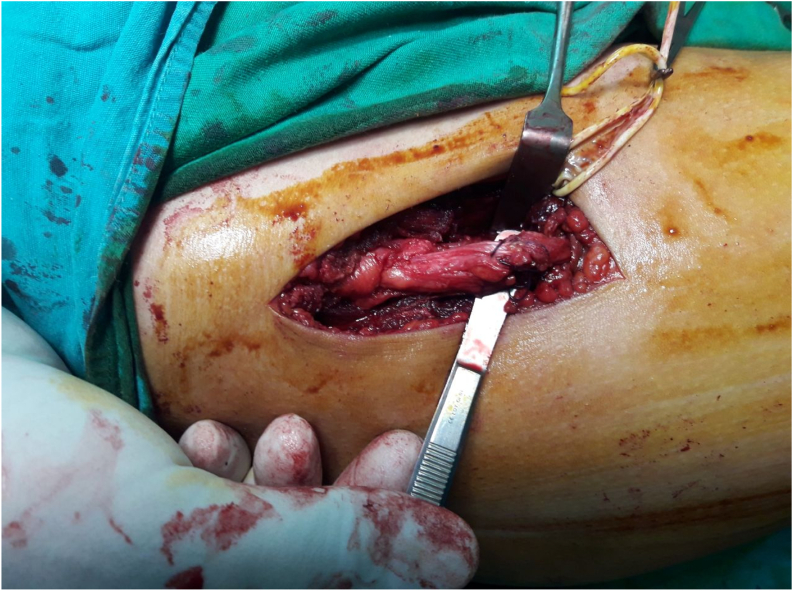
The rectus femoris muscle of the quadriceps femoris muscle after lengthening.

The patient underwent rectus femoris lengthening (RFL) via the anterolateral approach (Watson-Jones) to improve knee flexion. Under general anesthesia, the patient was positioned supine and an anterolateral skin incision was made over the proximal thigh. The subcutaneous tissue was dissected to expose the fascia lata, which was longitudinally incised. The lateral femoral cutaneous nerve was identified and protected. The rectus femoris tendon was isolated from the surrounding structures Fig. 3 and a Z-plasty was performed on the muscle tendon to achieve a 5 cm lengthening Fig. 4. The elongated ends of the muscle were approximated with a 1.0 polyglycolic suture. The fascia, subcutaneous tissue, and skin were closed in layers and a sterile dressing was applied. Intraoperative testing of knee flexion was performed by suspending the lower extremity from the operating table, stabilizing the pelvis, and flexing the knee, which demonstrated a full range of motion.

**Fig. 5:**
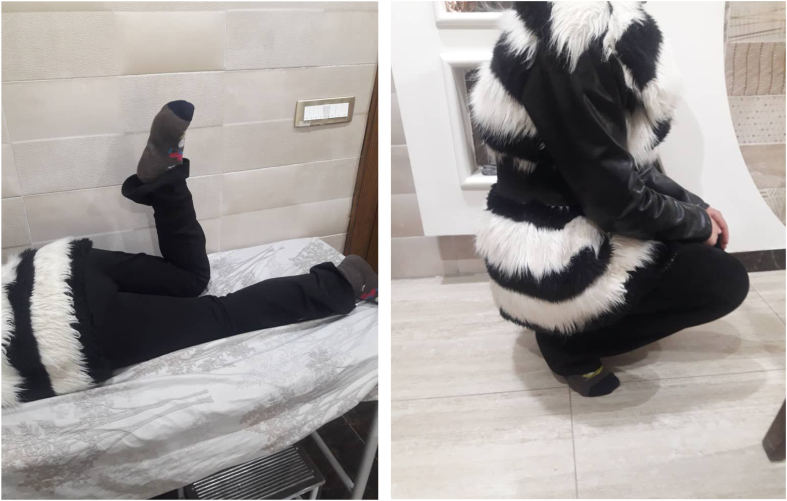
Clinical examination of the patient after follow-up.

Postoperatively, the patient was allowed early mobilization and physiotherapy, as well as ambulation with assistive devices. The sutures were removed on postoperative day 12 and physiotherapy was continued. The patient returned for follow-up after one month with complete knee flexion and hip extension. The patient was followed for three years with normal knee function and no complications Fig. 5.

## 3 Discussion

Quadriceps femoris muscle contracture in childhood can occur congenitally or as a result of various acquired factors such as iatrogenic injections in the thigh, trauma, infections, or ischemia [1,4]. In Syria, the prevalence of injection-induced contractures is high due to the widespread use of intramuscular injections for drug administration by healthcare practitioners [7]. In the case described, the patient did not have any congenital deformities in the lower extremities, but her parents reported a history of repeated intramuscular injections in the right thigh.

Quadriceps contracture leads to a stiff extended knee, with limited flexion depending on the severity of the contracture. Fibrosis and shortening of the muscle components, particularly the Vastus intermedius and Rectus femoris in the midline of the limb, contribute to the limitation of flexion [1,8].

There are two main manifestations of quadriceps femoris muscle contracture: isolated rectus femoris contracture, which limits knee flexion in hip extension but allows normal range of motion in hip flexion, and combined contracture of the quadriceps, which restricts knee flexion regardless of hip position [7]. Distinguishing between these two forms is crucial for determining the appropriate treatment approach. Ely's test plays a significant role in this differentiation, as it helps identify whether the quadriceps is fully contracted or if there is an isolated rectus femoris contracture. Distal release or lengthening is preferred for fully contracted quadriceps, while proximal release yields better outcomes for isolated rectus femoris contracture [7]. In the present case, the isolated rectus femoris form was identified. Additionally, another examination was conducted, where the patient was asked to lower her affected limb off the edge of the examination bed, which she could only do partially. This examination aided in ensuring full flexion of the knee during surgery.

Currently, there is no universally effective definitive procedure for all cases of quadriceps contracture, and various treatment methods have been recommended [4,7]. Therefore, each case must be individually assessed and managed. The selection of the appropriate surgical technique is crucial for improving outcomes and reducing recurrence rates. Postoperative physiotherapy is essential to support surgical outcomes and facilitate patient rehabilitation [7]. Conversely, preoperative physiotherapy and manipulation have limited effectiveness, except in mild and early cases, and may even pose risks such as supracondylar fractures [8]. Generally, passive stretching and physiotherapy are unlikely to yield long-lasting improvements [4].

In a study by Jackson and Hutton [3], 32 quadriceps contractures in 17 children were examined, comparing conservative management, distal quadricepsplasty, and proximal release. The best outcomes were observed with proximal release, while 70 % of cases undergoing distal quadricepsplasty experienced extensor lag, and the conservative group merely prevented further progression.

Several surgical techniques are employed for treatment, including quadriceps muscle tendon lengthening procedures like *Z*-tenotomy (“sliding Z-plasty”), V—Y plasty, and accordion techniques [9].

In our case, the patient underwent surgical treatment involving lengthening of the rectus femoris muscle using the Z-plasty procedure, which resulted in a 5 cm increase in muscle tendon length. This technique corresponds to a form of proximal release. The advantages of this approach lie in the significant increase in muscle length while preserving normal muscle function. Furthermore, it enables early ambulation without the need for immobilization, especially when combined with immediate postoperative physiotherapy and a splint was not used in this lengthening technique because the suture was well supported.

The most common complication encountered is incision necrosis. This is particularly true for anterior thigh skin incisions, which are under tension during knee flexion, especially in older children. To mitigate this problem, the incision should not extend distal to the superior pole of the patella. In some cases, a Z-plasty of the skin may be necessary to alleviate tension [8]. In our case, an incision was made proximal to the anterolateral aspect of the right thigh, which effectively prevented tension and minimized the occurrence of this complication.

In conclusion, injection-induced contracture of the rectus femoris muscle is a common condition in children due to repeated intramuscular injections in the thigh. However, healthcare practitioners need to be aware that medications can be administered in alternative sites such as the deltoid, triceps, or gluteal muscles, or through oral or intravenous routes if necessary.

## Consent for publication

Written informed consent was obtained from the patient's parents for the publication of this case report and any accompanying images. A copy of the written consent is available for review by the Editor-in-Chief of this journal.

## Ethical approval

Not required for case reports at our hospital. Single case reports are exempt from ethical approval in our institution.

## Funding

This research did not receive any specific grant from funding agencies in the public, commercial, or not-for-profit sectors.

## Author contribution

All authors were both involved in the conception and coordination of this report and drafted the manuscript. Additionally, all authors have read and approved the final version.

## Guarantor

Qusai Razzouk

## Research registration number

Not applicable

## Conflict of interest statement

No conflicts of interest.
